# *Cytochrome P450 1A1* gene polymorphisms and digestive tract cancer susceptibility: a meta‐analysis

**DOI:** 10.1111/jcmm.12853

**Published:** 2016-04-06

**Authors:** Anjing Ren, Tingting Qin, Qianqian Wang, Haina Du, Donghua Zhong, Yibing Hua, Lingjun Zhu

**Affiliations:** ^1^ Department of OncologyThe First Affiliated Hospital of Nanjing Medical UniversityNanjingChina; ^2^ Department of OncologyThe Third Affiliated Hospital of Nanjing University of T.C.MNanjingChina; ^3^ Department of General SurgeryThe First Affiliated Hospital of Nanjing Medical UniversityNanjingChina

**Keywords:** *CYP1A1*, digestive tract cancer, polymorphism, meta‐analysis

## Abstract

Cytochrome P450 1A1 (CYP1A1) is a phase I enzyme that regulates the metabolism of environmental carcinogens and alter the susceptibility to various cancers. Many studies have investigated the association between the *CYP1A1 MspI* and *Ile462Val* polymorphisms and digestive tract cancer (DTC) risk in different groups of populations, but their results were inconsistent. The PubMed and Embase Database were searched for case–control studies published up to 30th September, 2015. Data were extracted and pooled odds ratios (ORs) with 95% confidence intervals (CIs) were calculated to assess the relationship. Totally, 39 case–control studies (9094 cases and 12,487 controls) were included. The G allele in *Ile/Val* polymorphism was significantly associated with elevated DTC risk with per‐allele OR of 1.24 (95% CI = 1.09–1.41, *P* = 0.001). Similar results were also detected under the other genetic models. Evidence was only found to support an association between *MspI* polymorphism and DTC in the subgroups of caucasian and mixed individuals, but not in the whole population (the dominant model: OR = 1.19, 95% CI = 0.94–1.91, *P* = 0.146). In conclusion, our results suggest that the *CYP1A1* polymorphisms are potential risk factors for DTC. And large sample size and well‐designed studies with detailed clinical information are needed to more precisely evaluate our founding.

## Introduction

Digestive tract cancers (DTCs), well known as the most common malignant tumours globally, include oesophageal, gastric and colorectal cancers 1, 2, 3, 4. Data from *Global Cancer Statistics*, 2012 1 suggest that DTC has contributed to an enormous burden on society today. Actually, colorectal cancer is confirmed as the third most frequently diagnosed cancer in males and the second in females. Both the incidence rates of gastric cancer and oesophageal cancer keep the highest in Eastern Asia. Despite of the updating advances in surgery and chemotherapy, DTC remains the high‐mortality disease, even the leading cause of cancer‐related death 4. As generally accepted, the mechanism of the digestive tract tumorigenesis is a comprehensive combination of multiple risk factors including environmental conditions, dietary habits and genetic predispositions 5, 6, 7. Among these, metabolism‐associated genetic susceptibility has become an important focus. As a member of the CYP1 family, Cytochrome P4501A1 (CYP1A1) regulates the metabolism of many endogenous and exogenous carcinogens 3, 8, 9. *CYP1A1*, as its protein‐coding gene, is located on Chr15q22~q14, encoding aryl hydrocarbon hydroxylase. Aryl hydrocarbon hydroxylase is active in metabolizing some pro‐carcinogens, particularly the polycyclic aromatic hydrocarbons (PAHs), into intermediates. The intermediate substitutes may contribute to carcinogenesis eventually if bind to DNA and form adducts 10, 11, 12, 13, 14, 15.

*CYP1A1* gene consists of many single nucleotide polymorphisms (SNPs). These diverse variants could break the initial physiological equilibrium between activation and detoxification of metabolic carcinogens by adjusting the quantity and function of such enzyme. The two functional polymorphisms in *CYP1A1*gene, *MspI* (T >C, occurring in the noncoding 3′‐flanking region, rs4646903) and *Ile462Val* (A>G, found at codon 462 in exon 7, rs1048943), may associate with the risk of DTC by the mechanism above 9.

Many studies have been carried out to examine the association between the two polymorphisms of *CYP1A1* and risk of many cancers 9. However, because of different subject selections, the results were inconsistent. In addition, the relationship for DTC risk has been only explored in Chinese population by Liu *et al*. 14. Hence, to further explore that association in the whole of humanity and clarify the former results, we conduct this meta‐analysis with more eligible studies.

## Materials and methods

### Literature search strategy

The published case–control studies about the associations between the *CYP1A1* polymorphisms and DTC were searched manually on PubMed and Embase Database up to 30th September, 2015. The search was limited to English language papers, using the key words ‘(*CYP1A1* or *P4501A1* or *MspI* or exon7 or *Ile/Val* or cytochrome)’ and ‘polymorphism’ and ‘(colorectal cancer or gastric cancer or oesophageal cancer)’. And the following criteria were established: (*i*) case–control studies, (*ii*) exploring the association between *MspI* or *Ile/Val* polymorphism and DTC, (*iii*) DTC confirmed histologically or pathologically, (*iv*) providing sufficient data to calculate the odds ratio (OR) with its 95% confidence interval (CI) and *P*‐value. The exclusion criteria were as follows: (*i*) a case report or a review, (*ii*) no sufficient genotype frequency, (*iii*) a duplicated publication 10, 11, 12, 13, 14, 15.

### Data extraction

Based on the inclusion criteria listed above, two authors independently extracted data from all qualified publications. Controversies were eliminated through discussion with another investigator. Following data were collected: first author’ s name, year of publication, cancer type, country and ethnicity of population, genotyping method, source of controls, number of cases and controls with different genotypes, adjusted OR and 95% CI and adjustment of variables if available and Hardy–Weinberg equilibrium (HWE) 14, 15 (See in Tables 1 and 2).

**Table 1:** Characteristics of *CYP1A1 MspI* polymorphism included in the meta‐analysis

	Year	Ethnicity	Source	Case				Control				Method	Sample size	*P* for HWE	OR 95% CI^a^		Adjustment of variables
				*N*	Genotypes			*N*	Genotypes						CT/TT	CC/TT	
					TT	TC	CC		TT	TC	CC						
*MspI*																	
EC																	
Jain *et al*.	2007	Asian	PB	171	59	83	19	201	79	99	23	PCR	≥300	0.629	1.1 (0.71–1.7)	1.1 (0.55–2.2)	Age, gender, smoking, drinking
Malik *et al*.	2010	Asian	PB	135	76	52	7	195	95	88	12	MLPA	≥300	0.361	0.72 (0.45–1.14)	0.70 (0.26–1.87)	Age, gender
GC																	
Ma *et al*.	2006	Asian	PB	60	26	27	7	57	26	28	3	PCR‐RFLP	<300	0.423	–	–	–
Malik *et al*.	2009	Asian	HB	108	60	46	2	195	95	88	12	PCR	≥300	0.361	0.84 (0.52–1.37)	0.34 (0.07–1.60)	Age, gender
Luo *et al*.	2011	Asian	PB	123	38	61	24	129	47	54	28	PCR‐RFLP	<300	0.261	–	–	–
Ghoshal *et al*.	2014	Asian	PB	88	41	36	11	170	78	80	12	PCR‐RFLP	<300	0.370	–	–	–
Darazy *et al*.	2011	Caucasian	PB	11	9	0	2	56	54	1	1	PCR‐RFLP	<300	**0.000**	0.87 (0.5–1.5)	1.8 (0.7–4.4)	Age, gender
CC																	
Sivaraman *et al*.	1994	Mixed	PB	43	23	10	10	47	23	22	2	PCR‐RFLP	<300	0.508	–	–	–
Ye *et al*.	2002	Caucasian	NR	41	35	6	0	82	73	9	0	PCR‐RFLP	<300	0.871	–	–	–
Talseth *et al*.	2006	Caucasian	NR	118	94	20	4	100	91	9	0	PCR‐RFLP	<300	0.895	–	–	–
Darazy *et al*.	2011	Caucasian	PB	46	42	2	2	56	54	1	1	PCR‐RFLP	<300	**0.000**	–	–	–
Saeed *et al*.	2013	Asian	HB	94	70	21	3	79	73	6	0	PCR‐RFLP	<300	0.940	–	–	–
Rudolph *et al*.	2011	German	PB	679	539	134	6	679	564	102	13	KASPar assays	≥300	**0.007**	–	–	–

Significance of bold value: *P* < 0.05 for HWE is considered as significant disequilibrium.

a Adjusted. EC: oesophageal cancer; GC: gastric cancer; CC: colorectal cancer; HB: Hospital based; PB: Population based; NR: no record; HWE: Hardy–Weinberg equilibrium; PCR‐RFLP: polymerase chain reaction‐restriction fragment length polymorphism; PCR–ASO: PCR–allele specific oligonucleotide.

**Table 2:** Characteristics of *CYP1A1 Ile462Val* polymorphism included in the meta‐analysis

Study	Year	Ethnicity	Source	Case				Control				Method	Sample size	*P* for HWE	OR 95% CI^a^		Adjustment of variables
				*N*	Genotypes			*N*	Genotypes						GA/AA	GG/AA	
					AA	AG	GG		AA	AG	GG						
*Ile462Val*																	
EC																	
Morita *et al*.	1997	Asian	HB	53	32	20	1	132	80	49	3	PCR	<300	0.355	–	–	–
Nimura *et al*.	1997	Asian	HB	89	50	26	13	137	92	38	7	PTC‐150	<300	0.518	–	–	–
Hori *et al*.	1997	Asian	NR	101	62	37	2	428	275	133	20	nonRI‐SSCP	≥300	0.752	–	–	–
Lieshout *et al*.	1999	Caucasian	NR	34	26	8	0	247	207	37	3	PCR‐RFLP	<300	0.665	–	–	–
Wang *et al*.	2002	Asian	HB	127	25	58	44	101	31	48	22	PCR	<300	0.915	–	–	–
Wu *et al*.	2002	Asian	HB	146	68	62	16	324	179	127	18	PCR‐RFLP	≥300	0.762	1.34 (0.86–2.07)	2.48 (1.15–5.34)	Age, education, ethnicity, smoking, drinking, and areca consumption
Wang *et al*.	2003	Asian	PB	62	30	28	4	38	20	16	2	PCR‐RFLP	<300	0.870	–	–	–
Wang *et al*.	2004	Asian	HB	127	21	56	50	101	31	48	22	PCR	<300	0.915	1.7 (0.83–3.58)	3.3 (1.49–7.61)	Tobacco smoking、alcohol drinking、FHEC
Abbas *et al*.	2004	Caucasian	PB	79	61	9	9	130	101	6	23	PCR‐RFLP	<300	**0.000**	2.63 (0.84–8.28)	–	Age, sex
Wang *et al*.	2012	Asian	PB	565	304	225	36	468	295	154	19	PCR	≥300	0.981		–	–
Yun *et al*.	2013	Asian	PB	157	73	72	12	157	95	50	12	PCR‐RFLP	≥300	0.348	2.05 (1.19–3.54)	1.12 (0.41–3.04)	Age, gender, smoking, drinking and FHC
GC																	
Suzuki *et al*.	2004	Asian	HB	144	84	51	9	177	104	65	8	PCR	≥300	0.865		–	–
Li *et al*.	2005	Asian	HB	102	53	27	22	62	35	24	3	PCR	<300	0.910	0.59 (0.26–1.34)	5.91 (1.28–27.24)	Age, sex, education, job, drinking, smoking
Shen *et al*.	2005	Asian	PB	112	70	36	6	676	412	226	38	PCR‐RFLP	≥300	0.639	0.9 (0.5–1.4)	0.7 (0.2–1.8)	Age, gender, living areas, FHC, drinking
Agudo *et al*.	2006	Caucasian	PB	243	229	13	1	936	874	62	0	SNP500cd	≥300	0.578	0.90 (0.48–1.68)	–	Age, sex, centre, and date of blood extraction
Kobayashi *et al*.	2009	Asian	HB	141	91	44	6	286	162	109	15	MassARRAY system	≥300	0.832	0.79 (0.40–1.57)	2.01 (0.45–9.48)	*H. p* status, smoking, drink, FHGC, BMI, total food intake, JA membership
CC																	
Sivaraman *et al*.	1994	Mixed	PB	43	32	9	2	47	33	14	0	PCR‐RFLP	<300	0.487	–	–	–
Kiss *et al*.	2000	Mixed	PB	163	119	41	3	163	132	31	0	PCR‐ASO	≥300	0.407	–	–	–
Slattery *et al*.	2004	Mixed	HB	1791	1632	148	11	2180	1997	171	12	PCR	≥300	**0.001**	1.0 (0.7, 1.4)	1.5 (0.5, 4.9)	Age, sex
Little *et al*.	2006	Caucasian	PB	251	235	16	0	396	372	24	0	PCR	≥300	0.824	1.31 (0.59–2.91)	–	Age, sex, FHCC, aspirin use, use of other NSAIDs and physical activity
Yeh *et al*.	2007	Asian	HB	717	400	228	89	729	410	266	53	PCR‐RFLP	≥300	0.558	–	–	–
Yoshida *et al*.	2007	Asian	NR	66	34	27	5	121	79	37	5	PCR‐RFLP	<300	0.968	1.54 (0.78–3.04)	1.99 (0.41–9.63)	Age, gender, smoking habit
Pereira Serafim *et al*.	2008	Mixed	PB	114	14	97	3	114	81	33	0	PCR‐RFLP	<300	0.196	–	–	–
Kobayashi *et al*.	2009	Asian	HB	105	65	32	8	225	125	87	13	Mass ARRAY system	≥300	0.915	0.43 (0.171.06)	0.76 (0.144.13)	Smoking, drinking, FHCC, BMI, JA membership, and intake of other food
Nisa *et al*.	2010	Asian	PB	685	418	231	36	778	461	276	41	PCR‐RFLP	≥300	0.999	0.94 (0.75–1.17)	1.00 (0.62–1.62)	Age, sex, residence, smoking, drinking, BMI, job, physical activity, FHCC
Cleary *et al*.	2010	Caucasian	PB	1160	1052	98	10	1288	1166	114	8	Taq‐man	≥300	**0.023**	0.95 (0.71, 1.27)	1.37 (0.53, 3.55)	Age, sex

Significance of bold value: *P* < 0.05 for HWE is considered as significant disequilibrium.

a Adjusted. EC: oesophageal cancer; GC: gastric cancer; CC: colorectal cancer; HB: Hospital based; PB: Population based; NR: no record; HWE: Hardy–Weinberg equilibrium; PCR‐RFLP: polymerase chain reaction‐restriction fragment length polymorphism; PCR–ASO: PCR–allele specific oligonucleotide; FHEC: family history of EC.

### Statistical analysis

The HWE in control group was assessed by Pearson's goodness‐of‐fit chi‐square test and *P* < 0.05 was considered as significant disequilibrium. OR and 95% CI were calculated for *CYP1A1 MspI/Ile462Val* polymorphisms and DTC risk in each study. The pooled OR was also determined by the Z‐test (if *P* < 0.05, then considered as significant). Stratified analyses by cancer type, source of controls, ethnicity, sample size and genotyping method were performed 9, 10, 11, 12, 13, 14, 15.The influence of study size of each evaluated publication on the results was assessed by the weight.

Heterogeneity in our meta‐analysis was assessed by the chi‐square‐based Q‐test and *I* ^2^. A fixed‐effects model (the Mantel–Haenszel method) was applied if *P* > 0.05, which indicated no or little heterogeneity among eligible studies. Otherwise, the random‐effects model (Der Simonian and Laird method) was used. Galbraith graph was performed to explore the source of heterogeneity. Sensitivity analysis was tested to assess the stability of our results. The funnel plot was performed for potential publication bias. Funnel plot asymmetry was statistically assessed by Egger's linear regression test (publication bias exists if *P* < 0.05). All statistical analyses were carried out by Stata software (version 12.0, StataCorp LP, College Station, TX, USA) 13, 14, 15.

## Results

### Characteristics of studies

**Figure 1:**
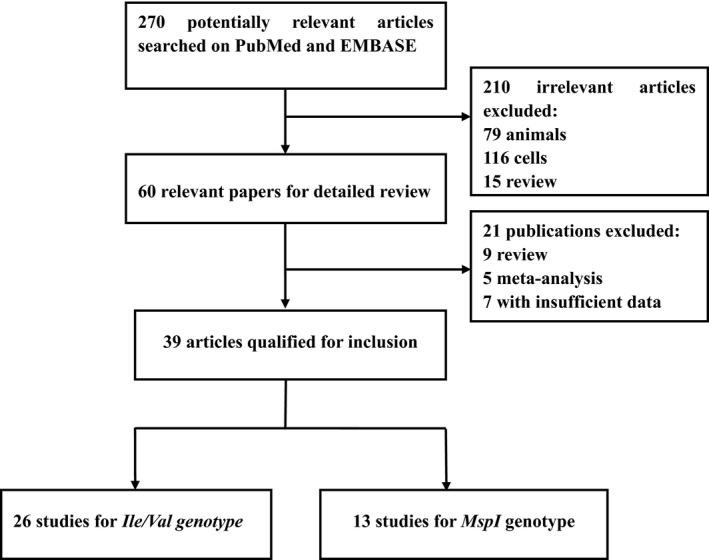
Studies identified with criteria for inclusion and exclusion.

Totally 37 publications 16, 17, 18, 19, 20, 21, 22, 23, 24, 25, 26, 27, 28, 29, 30, 31, 32, 33, 34, 35, 36, 37, 38, 39, 40, 41, 42, 43, 44, 45, 46, 47, 48, 49, 50, 51, 52 containing 39 studies (6 pieces not consistent with HWE were also included), which investigated the relationship between *CYP1A1* (*MspI* rs4646903 or *Ile/Val* rs1048943) polymorphisms and DTC risk, were included in the present meta‐analysis. The literature selection process was illustrated in Figure 1. All the eligible studies involved 9094 DTC cases and 12,487 controls. 13 studies (2 oesophageal cancer studies, 5 gastric cancer studies and 6 colorectal cancer studies) were identified for the *MspI* polymorphism, including a total of 1717 cases and 2046 controls. And for the *Ile/Val* polymorphism, 26 studies (11 oesophageal cancer studies, 5 gastric cancer studies and 10 colorectal cancer studies) were retrieved, covering a total of 7377 cases and 10,441 controls. More detailed charismatics about population source, ethnicity distribution, sample size, genotyping method and adjusted OR and 95% CI and adjustment of variables if available can be seen in Tables 1 and 2.

### Association of *MspI* with digestive tract cancer

**Figure 2:**
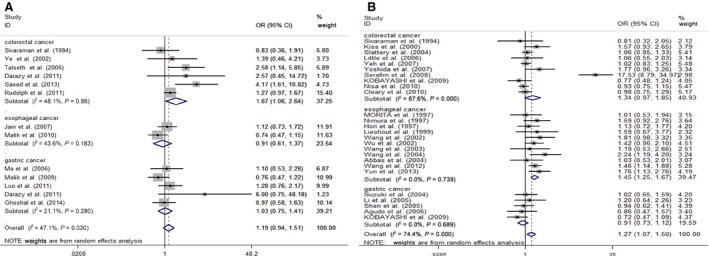
(**A**) Forest plot of digestive cancer risk associated with *MspI* polymorphism (the dominant model CC + CT *versus* TT). (**B**) Forest plot of digestive cancer risk associated with *Ile/Val* polymorphism (the dominant model GA+GG *versus* AA).

Overall, no sufficient evidence was found to support an association between increased susceptibility of DTC and *MspI* (rs4646903) polymorphism in all genetic models when all the eligible case–control studies were pooled together. Moreover, the adjusted pooled result was consistent with the crude one (data shown in Table 3 and Fig. 2A for the dominant model). In subgroup analysis by cancer type, a significant association was only found between *MspI* polymorphism and elevated colorectal cancer risk (the allele contrast: OR = 1.82, 95% CI = 1.16–2.86, *P* = 0.010). However, because of unavailable adjusted data on colorectal cancer, this positive result could not be validated (Fig. S1). Stratifying for ethnicity, an increased susceptibility was found in individuals with CC genotype among Caucasians and mixed population (the codominant model: OR = 1.39, 95% CI = 1.06–1.82, *P* = 0.018; OR = 5.7, 95% CI = 1.37–23.60, *P* = 0.016 respectively). However, no evidence was observed to prove that among Asians. In the stratified analysis by the source of controls, sample size or genotyping method, some statistical correlations were observed in the group of ‘population with sources unreported (NR)’, ‘size <300’ and ‘PCR‐RFLP method’ respectively (data shown in Table S1).

**Table 3:** The overall results for *MspI* and *Ile462Val* polymorphisms in *CYP1A1* and digestive tract cancer risk

		OR	95% CI	*P*	*I* ^ 2^ (%)	*Ph*	OR^a^	95% CI^a^	*P* a	*I* ^ 2^ (%)^a^	*Ph* a
*MspI*											
Allele	C/T	1.24	0.99–1.54	0.058	59.60%	0.003	–	–	–		
Dominant	CC+CT/TT	1.19	0.94–1.91	0.146	47.10%	0.030	–	–	–		
Resessive	CC/CT+TT	1.32	0.80–2.17	0.283	49.50%	0.026	–	–	–		
Codominant	CT/TT	1.12	0.88–1.42	0.341	42.00%	0.055	0.88	0.69–1.12	0.296	0.0%	0.624
	CC/TT	1.30	0.80–1.21	0.296	43.50%	0.053	1.01	0.64–1.62	0.937	24.4%	0.265
*Ile462Val*											
Allele	G/A	1.24	1.09–1.41	**0.001**	69.40%	0.000	–	–	–		
Dominant	GA+GG/AA	1.27	1.07–1.50	**0.006**	74.40%	0.000	–	–	–		
Resessive	GG/AA+GA	1.49	1.21–1.82	**0.000**	22.30%	0.157	–	–	–		
Codominant	GA/AA	1.21	1.02–1.45	**0.032**	74.20%	0.000	1.03	0.92–1.67	0.593	37.9%	0.074
	GG/AA	1.58	1.24–2.00	**0.000**	35.40%	0.042	1.49	1.23–1.96	**0.005**	30.1%	0.160

Significance of bold value: *P* < 0.05 means a significant relationship between the polymorphism and digestive tract cancer risk.

a Adjusted. *Ph*:* P*‐value of Q‐test for heterogeneity identification; *I* ^2^ index: a quantitative measurement which indicates the proportion of total variation in study estimates that is due to between‐study heterogeneity.

### Association of *Ile/Val* with digestive tract cancer

The G allele was significantly associated with elevated DTCs risk with per‐allele OR of 1.24 (95% CI = 1.09–1.41, *P* = 0.001). Similar results were also detected under other genetic models and in our adjusted pooled result (data shown in Table 3 and Fig. 2B, the dominant model). In the further subgroup analysis based on tumour type, the statistics strongly supported the significant relationship between *Ile/Val* and the chance of suffering oesophageal and colorectal cancer (the allele contrast: OR = 1.36, 95% CI = 1.19–1.56, *P* = 0.000, OR = 1.27, 95% CI = 1.01–1.61, *P* = 0.043 respectively). But the positive result was only observed in oesophagus cancer from the adjusted result partially together (Fig. S2). A significant association was also observed in Asians (the codominant model: OR = 1.62, 95% CI = 1.26–2.09, *P* = 0.000), but not in caucasians or mixed individuals. Stratified by the source of controls, significant association was observed both in HB and NR group. Stratified by sample size and genotyping method, associations were found in most groups. Detailed analyses of the genetic variant are provided in Table S2.

### Heterogeneity analyses

**Figure 3:**
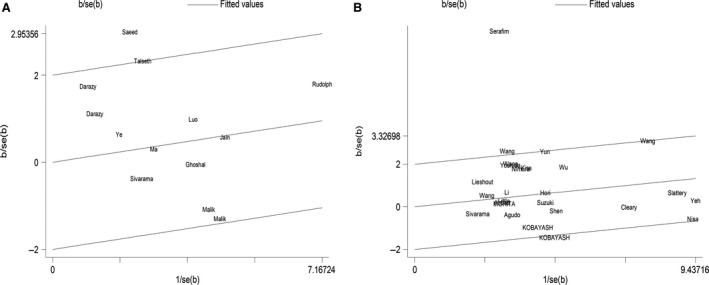
(**A**) Galbraith graph for *MspI* polymorphism (the dominant model CC + CT *versus* TT): the study conducted by Saeed *et al*. may be the main source of heterogeneity. (**B**) Galbraith graph for *Ile/Val* polymorphism (the dominant model GA+GG *versus* AA): the study conducted by Pereira Serafim *et al*. may be the main source of heterogeneity.

For *MspI* polymorphism, moderate heterogeneity was detected (*e.g*. the dominant model: *I* ^2^ = 47.1%, *Ph* = 0.030). As shown in Tables S3 and S4, subgroup analyses stratified by cancer type, ethnicity, source of controls, sample size and genotyping method could not explain the source of heterogeneity at length. Hence, to further explore the heterogeneity source, we performed Galbraith graph. The study conducted by Saeed *et al*. 24 may be the main source of heterogeneity (data shown in Fig. 3A). Removing this study, the result of the meta‐analysis did not change essentially (*e.g*. the dominant mode: OR = 1.10, 95% CI = 0.90–1.35, *P* = 0.336), but its heterogeneity decreased significantly (the dominant model: *I* ^2^ = 28.6%, *Ph* = 0.165) (Fig. S3). Similar results were observed in other genetic models. In the same way, we found the source of heterogeneity in Figure 3B for *Ile/Val* polymorphism. When we removed the study conducted by Pereira Serafim *et al*. 47, the heterogeneity decreased sharply, while the results remained qualitatively (the dominant mode: OR = 1.14, 95% CI = 1.03–1.27, *P* = 0.016; *I* ^2^ = 34.8%, *Ph* = 0.046) (Fig. S4).

### Sensitivity analyses

**Figure 4:**
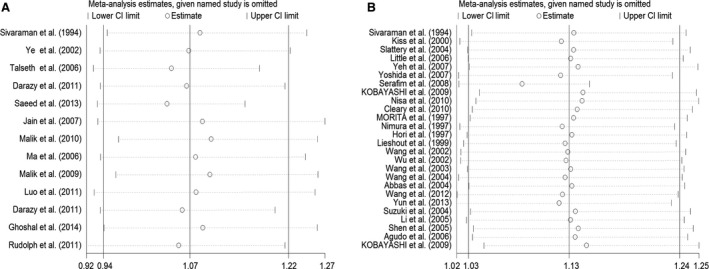
(**A**) Influence analysis of the summary odds ratio coefficients on the association between *MspI* polymorphism with digestive tract cancers risk (the dominant model CC + CT *versus* TT). Results were computed by omitting each study (left column) in turn. Bars, 95% CI. (**B**) Influence analysis of the summary odds ratio coefficients on the association between *Ile/Val* polymorphism with digestive tract cancers risk (the dominant model GA + GG *versus* AA). Results were computed by omitting each study (left column) in turn. Bars, 95% CI.

The corresponding pooled ORs were not qualitatively influenced when any particular study had been removed from the meta‐analysis (including the studies not conforming to HWE) for the two polymorphisms respectively (see in Fig. 4A and B). It confirmed that the results of the present meta‐analysis are reliable and stable.

### Publication Bias

**Figure 5:**
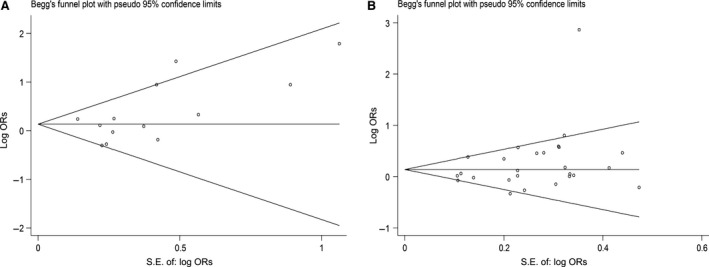
(**A**) Begg's funnel plot for publication bias test for *MspI* polymorphism (the dominant model CC + CT *versus* TT). Each point represents a separate study for the indicated association. (**B**) Begg's funnel plot for publication bias test *Ile/Val* polymorphism (the dominant model GA+GG *versus* AA). Each point represents a separate study for the indicated association.

Begg's funnel plot and Egger's test were performed to diagnose the publication bias of papers. The shapes of the funnel plots did not reveal any evidence of obvious asymmetry in all comparison models for *MspI* (*e.g*. the dominant model in Fig. 5A). Statistically the results of both tests showed no publication bias (Begg's test *P* = 0.127, Egger's test *P* = 0.136, *t* = 1.61, 95% CI = −0.46 to 2.97). Regarding *Ile/Val*, no publication bias was detected as well in the dominant model (Begg's test *P* = 0.071, Egger's test *P* = 0.085, *t* = 1.80, 95% CI = −0.23 to 3.30) (Fig. 5B).

## Discussion

CYP1A, the subfamily of Cytochrome P450, is an important phase I metabolic enzyme. As a key subtype of CYP1A, CYP1A1 is distributed widely in the kidney, lung, stomach, colon, larynx, placenta, skin, lymphocyte, brain and other tissues 14. What's more, recent studies have demonstrated that it involves the metabolism of some exogenous carcinogens such as PAHs. *CYP1A1* gene can promote the carcinogenic process by converting PAHs into their ultimate DNA‐binding forms 11.

*MspI* and *Ile/Val*, the main gene polymorphisms of *CYP1A1*, have been both verified associated with many kinds of cancers by large number of meta‐analyses 9. However, inconsistent results have been reported. To clarify this inconsistency, this meta‐analysis was established. To our best knowledge, it is the first one to explore the association of *CYP1A1* polymorphisms and DTC risk in the whole population. Correlation association between *CYP1A1 Ile/Val* polymorphism and DTC susceptibility were detected in our meta‐analysis. While no evidence showed the association between *CYP1A1 MspI* polymorphism and DTC risk. The overall result is consistent with that of the meta‐analysis performed by Liu *et al*. 14 which was designed only in Chinese population.

Stratified by cancer type, the *MspI* CC genotype carriers were confirmed with an increased susceptibility to colorectal cancer but not to oesophageal or gastric cancer. While an A to G mutation in *Ile/Val* polymorphism increased the cancer risk in EC and CC groups. The results were partially inconsistent with Wu *et al*. 9. In fact, the studies we included in the present meta‐analysis were updated compared with Wu *et al*. And unhealthy eating habits could contribute to the digestive tract damage, such as excessive drinking. That is why different primary cancers of digestive tract may be caused by similar risk factors 13. On the other hand, DTC includes so many kinds of malignant tumours that heterogeneities among them will be found. One reason for the issue may be that the gene–gene and gene–environment interactions mechanisms differ in diverse digestive tract parts. To our common knowledge, some of the digestive tract tumours have their specific risk factors. For instance eating spicy and hot food can evaluate the risk of oesophageal cancer, whereas diet with high fat and low fibre may enhance the incidence of colorectal cancer. In addition, researchers have verified that *Helicobacter pylori* infection significantly increased susceptibility to gastric cancer 5, 6, 13, 18. In a word, the aetiological factors sensitive to various types of DTCs are not all the same. In the subgroup analysis by ethnicity, significant difference was detected in caucasian and mixed group for *MspI* polymorphism. Interestingly, high correlativity was otherwise observed in Asian group for *Ile/Val* polymorphism. This think‐provoking phenomenon may excellently reveal that genetic diversity exactly exists among various ethnicities across countrywide. Individuals, disturbing in different places of the world, will experience different environments, including climate, temperature and radiation 7 and will form diverse lifestyles especially a variety of eating habits. Both of the above will contribute to the genetic background discrepancy among ethnicities. In addition, we conduct two subgroup analyses for adjusted status (Yes or no) and adjusted status especially for smoking history (Yes or no) for *Ile/Val* polymorphism. The result in every subgroup is corresponding (Table S5), which verified the reliability of our results again. As the number of studies with adjusted data for *MspI* polymorphism is only 4, and moreover, only one study provided adjusted data for smoking, we did not carry out the analyses for *MspI* polymorphism.

Some limitations and potential bias cannot be ignored in our meta‐analysis. First, we centre on the heterogeneity. Moderate and high heterogeneity were detected among the studies for *MspI* and *Ile/Val* respectively. Through Galbraith graph, we found the study conducted by Saeed *et al*. 24 count for the main source of heterogeneity for *MspI*. For *Ile/Val*, the heterogeneity mainly came from study conducted by Pereira Serafim *et al*. 47. Through reviewing the two papers, we found some reasons to explain that. In the former study, the population was from Saudi Arabia and the number of case and control group is 94/79. While in the later, the population was from Brazil and the number of case and control group is 114/114. In our point, both Saudi Arabia and Brazil have vast territories and long histories. Hence, maybe the ethnic origins are complex. And the lifestyles and customs may vary significantly across the two countries, respectively, which would contribute to great heterogeneity. What is more, the sample sizes of both studies are relatively smaller. Concluding from the results of subgroup analyses, the sample size, the source of controls and the genotyping method also influence the heterogeneity in a certain degree. Thus, more studies with large enough sample sizes and more detailed criteria are warranted. Lastly, published studies were included in our studies, whereas many other unpublished data were ignored. Therefore, potentially publication bias will exist in our study.

In summary, our meta‐analysis revealed the significant association between the *CYP1A1 Ile462Val* polymorphism and increased digest tract cancers risk. While no sufficient evidence was found to support the association between the *CYP1A1 MspI* polymorphism and DTC. In the subgroup analyses, the positive results were found in CC group, caucasians and mixed individuals for *MspI* polymorphism. Our results suggest that the *CYP1A1* polymorphisms are potential risk factors for DTC. Large sample size and well‐designed studies with more clinical information like age, gender, smoking and drinking are needed to clarify our finding.

## Conflict of interest

The authors declare no competing financial interest.

## Author contribution

LJZ and YBH conceived and designed the study. HND and AJR performed the experiments. AJR, TTQ, QQW and DHZ analysed the data. AJR, TTQ, QQW and DHZ contributed to the reagents/materials/analysis tools. AJR wrote the manuscript. All authors reviewed the manuscript.

## Supporting information


**Figure S1** Forest plot of digestive cancer risk associated with *MspI* polymorphism with adjusted OR and 95% CI (the codominant model CC *versus* TT).
**Figure S2** Forest plot of digestive cancer risk associated with *Ile*/*Val* polymorphism with adjusted OR and 95% CI (the codominant model GG *versus* AA).
**Figure S3** Forest plot of digestive cancer risk associated with *MspI* polymorphism after dropping the data from Saeed *et al*. 2013^24^ (the dominant model CC + CT *versus* TT).
**Figure S4** Forest plot of digestive cancer risk associated with *Ile*/*Val* polymorphism after dropping the data from Pereira Serafim *et al*. 2008 47 (the dominant model GA+GG *versus* AA).
**Table S1** Pooled ORs and 95% CIs of stratified meta‐analysis for *MspI* polymorphism.
**Table S2** Pooled ORs and 95% CIs of stratified meta‐analysis for *Ile*/*Val* polymorphism.
**Table S3** Heterogeneity test for *MspI* polymorphism.
**Table S4** Heterogeneity test for *Ile*/*Val* polymorphism.
**Table S5** Subgroup analyses for adjusted status (Yes or no) and adjusted status especially for smoking history (Yes or no) for Ile/Val polymorphism (GG/AA model).

## References

[1] Torre LA , Bray F , Siegel RL , *et al* Global cancer statistics, 2012. CA Cancer J Clin. 2015; 65: 87–108. 25651787 10.3322/caac.21262

[2] Peleteiro B , Castro C , Morais S , *et al* Worldwide burden of gastric cancer attributable to tobacco smoking in 2012 and predictions for 2020. Dig Dis Sci. 2015; 60: 2470–6. 25786860 10.1007/s10620-015-3624-x

[3] Lakhoo K . Neonatal surgical conditions. Early Hum Dev. 2014; 90: 933. 25448784 10.1016/j.earlhumdev.2014.09.015

[4] Zhang JM , Cui XJ , Xia YQ , *et al* Correlation between *TGF‐beta1‐509 C>T* polymorphism and risk of digestive tract cancer in a meta‐analysis for 21,196 participants. Gene. 2012; 505: 66–74. 22677269 10.1016/j.gene.2012.05.046

[5] Zhu CL , Huang Q , Liu CH , *et al* *NAD(P)H: quinone oxidoreductase 1 (NQO1) C609T* gene polymorphism association with digestive tract cancer: a meta‐analysis. Asian Pac J Cancer Prev. 2013; 14: 2349–54. 23725139 10.7314/apjcp.2013.14.4.2349

[6] Yao L , Wang HC , Liu JZ , *et al* Quantitative assessment of the influence of *cytochrome P450 2C19* gene polymorphisms and digestive tract cancer risk. Tumour Biol. 2013; 34: 3083–91. 23754447 10.1007/s13277-013-0875-z

[7] Zhang X , Zhang Y , Gu D , *et al* Increased risk of developing digestive tract cancer in subjects carrying the *PLCE1 rs2274223 A>G* polymorphism: evidence from a meta‐analysis. PLoS ONE. 2013; 8: e76425. 24116107 10.1371/journal.pone.0076425 PMC3792074

[8] Li P , Xu J , Shi Y , *et al* Association between zinc intake and risk of digestive tract cancers: a systematic review and meta‐analysis. Clin Nutr. 2014; 33: 415–20. 24148607 10.1016/j.clnu.2013.10.001

[9] Wu B , Liu K , Huang H , *et al* *MspI* and *Ile462Val* polymorphisms in *CYP1A1* and overall cancer risk: a meta‐analysis. PLoS ONE. 2013; 8: e85166. 24391993 10.1371/journal.pone.0085166 PMC3877352

[10] Gong FF , Lu SS , Hu CY , *et al* *Cytochrome P450 1A1* (*CYP1A1*) polymorphism and susceptibility to esophageal cancer: an updated meta‐analysis of 27 studies. Tumour Biol. 2014; 35: 10351–61. 25048966 10.1007/s13277-014-2341-y

[11] Yu KT , Ge C , Xu XF , *et al* *CYP1A1* polymorphism interactions with smoking status in oral cancer risk: evidence from epidemiological studies. Tumour Biol. 2014; 35: 11183–91. 25106409 10.1007/s13277-014-2422-y

[12] Meng FD , Ma P , Sui CG , *et al* Association between *cytochrome P450 1A1 (CYP1A1)* gene polymorphisms and the risk of renal cell carcinoma: a meta‐analysis. Sci Rep. 2015; 5: 8108. 25630554 10.1038/srep08108 PMC4309971

[13] Du H , Guo N , Shi B , *et al* The effect of *XPD* polymorphisms on digestive tract cancers risk: a meta‐analysis. PLoS ONE. 2014; 9: e96301. 24787743 10.1371/journal.pone.0096301 PMC4008560

[14] Liu C , Jiang Z , Deng QX , *et al* Meta‐analysis of association studies of *CYP1A1* genetic polymorphisms with digestive tract cancer susceptibility in Chinese. Asian Pac J Cancer Prev. 2014; 15: 4689–95. 24969905 10.7314/apjcp.2014.15.11.4689

[15] Chen B , Cao L , Yang P , *et al* *Cyclin D1 (CCND1) G870A* gene polymorphism is an ethnicity‐dependent risk factor for digestive tract cancers: a meta‐analysis comprising 20,271 subjects. Cancer Epidemiol. 2012; 36: 106–15. 21606015 10.1016/j.canep.2011.04.007

[16] Sivaraman L , Leatham MP , Yee J , *et al* *CYP1A1* genetic polymorphisms and *in situ* colorectal cancer. Cancer Res. 1994; 54: 3692–5. 7913406

[17] Ye Z , Parry JM . Genetic polymorphisms in the *cytochrome P450 1A1*,* glutathione S‐transferase M1 and T1*, and susceptibility to colon cancer. Teratog Carcinog Mutagen. 2002; 22: 385–92. 12210502 10.1002/tcm.10035

[18] Ma JX , Zhang KL , Liu X , *et al* Concurrent expression of aryl hydrocarbon receptor and *CYP1A1* but not *CYP1A1 MspI* polymorphism is correlated with gastric cancers raised in Dalian. China. Cancer Lett. 2006; 240: 253–60. 16337337 10.1016/j.canlet.2005.09.020

[19] Talseth BA , Meldrum C , Suchy J , *et al* Genetic polymorphisms in xenobiotic clearance genes and their influence on disease expression in hereditary nonpolyposis colorectal cancer patients. Cancer Epidemiol Biomarkers Prev. 2006; 15: 2307–10. 17119063 10.1158/1055-9965.EPI-06-0040

[20] Luo YP , Chen HC , Khan MA , *et al* Genetic polymorphisms of metabolic enzymes*‐CYP1A1, CYP2D6, GSTM1, and GSTT1*, and gastric carcinoma susceptibility. Tumour Biol. 2011; 32: 215–22. 20878561 10.1007/s13277-010-0115-8

[21] Darazy M , Balbaa M , Mugharbil A , *et al* *CYP1A1, CYP2E1, and GSTM1* gene polymorphisms and susceptibility to colorectal and gastric cancer among Lebanese. Genet Test Mol Biomarkers. 2011; 15: 423–9. 21385088 10.1089/gtmb.2010.0206

[22] Rudolph A , Sainz J , Hein R , *et al* Modification of menopausal hormone therapy‐associated colorectal cancer risk by polymorphisms in sex steroid signaling, metabolism and transport related genes. Endocr Relat Cancer. 2011; 18: 371–84. 21490239 10.1530/ERC-11-0057

[23] Ghoshal U , Tripathi S , Kumar S , *et al* Genetic polymorphism of *cytochrome P450 (CYP) 1A1, CYP1A2, and CYP2E1* genes modulate susceptibility to gastric cancer in patients with *Helicobacter pylori* infection. Gastric Cancer. 2014; 17: 226–34. 23686565 10.1007/s10120-013-0269-3

[24] Saeed HM , Alanazi MS , Nounou HA , *et al* *Cytochrome P450 1A1, 2E1 and GSTM1* gene polymorphisms and susceptibility to colorectal cancer in the Saudi population. Asian Pac J Cancer Prev. 2013; 14: 3761–8. 23886179 10.7314/apjcp.2013.14.6.3761

[25] Jain M , Kumar S , Ghoshal UC , *et al* *CYP1A1 Msp1 T/C* polymorphism in esophageal cancer: no association and risk modulation. Oncol Res. 2007; 16: 437–43. 18074679 10.3727/000000007783980846

[26] Malik MA , Upadhyay R , Mittal RD , *et al* Association of xenobiotic metabolizing enzymes genetic polymorphisms with esophageal cancer in Kashmir Valley and influence of environmental factors. Nutr Cancer. 2010; 62: 734–42. 20661821 10.1080/01635581003605904

[27] Morita S , Yano M , Shiozaki H , *et al* *CYP1A1, CYP2E1 and GSTM1* polymorphisms are not associated with susceptibility to squamous‐cell carcinoma of the esophagus. Int J Cancer. 1997; 71: 192–5. 9139841 10.1002/(sici)1097-0215(19970410)71:2<192::aid-ijc11>3.0.co;2-k

[28] Nimura Y , Yokoyama S , Fujimori M , *et al* Genotyping of the *CYP1A1 and GSTM1* genes in esophageal carcinoma patients with special reference to smoking. Cancer. 1997; 80: 852–7. 9307183 10.1002/(sici)1097-0142(19970901)80:5<852::aid-cncr4>3.0.co;2-n

[29] Hori H , Kawano T , Endo M , *et al* Genetic polymorphisms of tobacco‐ and alcohol‐related metabolizing enzymes and human esophageal squamous cell carcinoma susceptibility. J Clin Gastroenterol. 1997; 25: 568–75. 9451664 10.1097/00004836-199712000-00003

[30] van Lieshout EM , Roelofs HM , Dekker S , *et al* Polymorphic expression of *the glutathione S‐transferase P1* gene and its susceptibility to Barrett's esophagus and esophageal carcinoma. Cancer Res. 1999; 59: 586–9. 9973204

[31] Wang AH , Sun CS , Li LS , *et al* Relationship of tobacco smoking *CYP1A1 GSTM1* gene polymorphism and esophageal cancer in Xi'an. World J Gastroenterol. 2002; 8: 49–53. 11833070 10.3748/wjg.v8.i1.49 PMC4656624

[32] Wu MT , Lee JM , Wu DC , *et al* Genetic polymorphisms of *cytochrome P4501A1* and oesophageal squamous‐cell carcinoma in Taiwan. Br J Cancer. 2002; 87: 529–32. 12189551 10.1038/sj.bjc.6600499 PMC2376151

[33] Wang LD , Zheng S , Liu B , *et al* *CYP1A1, GSTs and mEH* polymorphisms and susceptibility to esophageal carcinoma: study of population from a high‐ incidence area in north China. World J Gastroenterol. 2003; 9: 1394–7. 12854128 10.3748/wjg.v9.i7.1394 PMC4615470

[34] Wang AH , Sun CS , Li LS , *et al* Genetic susceptibility and environmental factors of esophageal cancer in Xi'an. World J Gastroenterol. 2004; 10: 940–4. 15052670 10.3748/wjg.v10.i7.940 PMC4717108

[35] Abbas A , Delvinquiere K , Lechevrel M , *et al* *GSTM1, GSTT1, GSTP1 and CYP1A1* genetic polymorphisms and susceptibility to esophageal cancer in a French population: different pattern of squamous cell carcinoma and adenocarcinoma. World J Gastroenterol. 2004; 10: 3389–93. 15526353 10.3748/wjg.v10.i23.3389 PMC4576215

[36] Wang D , Su M , Tian D , *et al* Associations between *CYP1A1 and CYP2E1* polymorphisms and susceptibility to esophageal cancer in Chaoshan and Taihang areas of China. Cancer Epidemiol. 2012; 36: 276–82. 22088806 10.1016/j.canep.2011.10.008

[37] Yun YX , Wang YP , Wang P , *et al* *CYP1A1* genetic polymorphisms and risk for esophageal cancer: a case‐control study in central China. Asian Pac J Cancer Prev. 2014; 14: 6507–12. 24377558 10.7314/apjcp.2013.14.11.6507

[38] Kiss I , Sandor J , Pajkos G , *et al* Colorectal cancer risk in relation to genetic polymorphism of cytochrome *P450 1A1, 2E1,* and glutathione‐S‐transferase M1 enzymes. Anticancer Res. 2000; 20: 519–22. 10769717

[39] Suzuki S , Muroishi Y , Nakanishi I , *et al* Relationship between genetic polymorphisms of drug‐metabolizing enzymes (CYP1A1, CYP2E1, GSTM1, and NAT2), drinking habits, histological subtypes, and *p53* gene point mutations in Japanese patients with gastric cancer. J Gastroenterol. 2004; 39: 220–30. 15064998 10.1007/s00535-003-1281-x

[40] Slattery ML , Samowtiz W , Ma K , *et al* *CYP1A1*, cigarette smoking, and colon and rectal cancer. Am J Epidemiol. 2004; 160: 842–52. 15496536 10.1093/aje/kwh298

[41] Li H , Chen XL , Li HQ . Polymorphism of *CYPIA1 and GSTM1* genes associated with susceptibility of gastric cancer in Shandong Province of China. World J Gastroenterol. 2005; 11: 5757–62. 16270381 10.3748/wjg.v11.i37.5757 PMC4479672

[42] Shen J , Wang RT , Xu YC , *et al* Interaction models of *CYP1A1, GSTM1* polymorphisms and tobacco smoking in intestinal gastric cancer. World J Gastroenterol. 2005; 11: 6056–60. 16273625 10.3748/wjg.v11.i38.6056 PMC4436735

[43] Little J , Sharp L , Masson LF , *et al* Colorectal cancer and genetic polymorphisms of *CYP1A1, GSTM1 and GSTT1*: a case‐control study in the Grampian region of Scotland. Int J Cancer. 2006; 119: 2155–64. 16823842 10.1002/ijc.22093

[44] Agudo A , Sala N , Pera G , *et al* Polymorphisms in metabolic genes related to tobacco smoke and the risk of gastric cancer in the European prospective investigation into cancer and nutrition. Cancer Epidemiol Biomarkers Prev. 2006; 15: 2427–34. 17164366 10.1158/1055-9965.EPI-06-0072

[45] Yeh CC , Sung FC , Tang R , *et al* Association between polymorphisms of biotransformation and DNA‐repair genes and risk of colorectal cancer in Taiwan. J Biomed Sci. 2007; 14: 183–93. 17191090 10.1007/s11373-006-9139-x

[46] Yoshida K , Osawa K , Kasahara M , *et al* Association of *CYP1A1, CYP1A2, GSTM1 and NAT2* gene polymorphisms with colorectal cancer and smoking. Asian Pac J Cancer Prev. 2007; 8: 438–44. 18159984

[47] Pereira Serafim PV , Cotrim Guerreiro da Silva ID , Manoukias Forones N . Relationship between genetic polymorphism of *CYP1A1 at codon 462 (Ile462Val)* in colorectal cancer. Int J Biol Markers. 2008; 23: 18–23. 18409146 10.1177/172460080802300103

[48] Kobayashi M , Otani T , Iwasaki M , *et al* Association between dietary heterocyclic amine levels, genetic polymorphisms of *NAT2, CYP1A1, and CYP1A2* and risk of colorectal cancer: a hospital‐based case‐control study in Japan. Scand J Gastroenterol. 2009; 44: 952–9. 19452301 10.1080/00365520902964721

[49] Kobayashi M , Otani T , Iwasaki M , *et al* Association between dietary heterocyclic amine levels, genetic polymorphisms of *NAT2, CYP1A1, and CYP1A2* and risk of stomach cancer: a hospital‐based case‐control study in Japan. Gastric Cancer. 2009; 12: 198–205. 20047124 10.1007/s10120-009-0523-x

[50] Nisa H , Kono S , Yin G , *et al* Cigarette smoking, genetic polymorphisms and colorectal cancer risk: the Fukuoka Colorectal Cancer Study. BMC Cancer. 2010; 10: 274. 20534171 10.1186/1471-2407-10-274 PMC2906477

[51] Cleary SP , Cotterchio M , Shi E , *et al* Cigarette smoking, genetic variants in carcinogen‐metabolizing enzymes, and colorectal cancer risk. Am J Epidemiol. 2010; 172: 1000–14. 20937634 10.1093/aje/kwq245 PMC2984254

[52] Malik MA , Upadhyay R , Mittal RD , *et al* Role of xenobiotic‐metabolizing enzyme gene polymorphisms and interactions with environmental factors in susceptibility to gastric cancer in Kashmir Valley. J Gastrointest Cancer. 2009; 40: 26–32. 19521675 10.1007/s12029-009-9072-0

