# Supplementary material for: Cytochrome P450 1A1 gene polymorphisms and digestive tract cancer susceptibility: a meta‐analysis
Source: J Cell Mol Med. 2016 Apr 6;20(9):1620–31. doi: 10.1111/jcmm.12853 (PMC4988294; doi:10.1111/jcmm.12853)
Supplement: Supplementary file 1 — Figure S1 Forest plot of digestive cancer risk associated with MspI polymorphism with adjusted OR and 95% CI (the codominant model CC versus TT). Figure S2 Forest plot of digestive cancer risk associated with Ile/Val polymorphism with adjusted OR and 95% CI (the codominant model GG versus AA). Figure S3 Forest plot of digestive cancer risk associated with MspI polymorphism after dropping the data from Saeed et al. 201324 (the dominant model CC + CT versus TT). Figure S4 Forest plot of digestive cancer risk associated with Ile/Val polymorphism after dropping the data from Pereira Serafim et al. 2008 47 (the dominant model GA+GG versus AA). Table S1 Pooled ORs and 95% CIs of stratified meta‐analysis for MspI polymorphism. Table S2 Pooled ORs and 95% CIs of stratified meta‐analysis for Ile/Val polymorphism. Table S3 Heterogeneity test for MspI polymorphism. Table S4 Heterogeneity test for Ile/Val polymorphism. Table S5 Subgroup analyses for adjusted status (Yes or no) and adjusted status especially for smoking history (Yes or no) for Ile/Val polymorphism (GG/AA model). [file JCMM-20-1620-s001.docx]

***[Cytochrome P450 1A1](http://www.ncbi.nlm.nih.gov/pubmed/22733497)* Gene Polymorphisms and Digestive Tract Cancer Susceptibility: A Meta-Analysis**

Anjing Ren^a#^, Tingting Qin^a#^, Qianqian Wang^a#^, Haina Du^b^, Donghua Zhong^c^, Yibing Hua^c^*, Lingjun Zhu^a^*
^a^ Department of Oncology, The First Affiliated Hospital of Nanjing Medical University, Nanjing, China

^b^ Department of Oncology, The Third Affiliated Hospital of Nanjing University of T.C.M, Nanjing, China

^c^ Department of General Surgery, The First Affiliated Hospital of Nanjing Medical University, Nanjing, China

^#^ These authors contributed equally to this work.

* Corresponding author:

Lingjun Zhu: Department of Oncology, The First Affiliated Hospital of Nanjing Medical University, 300 GuangZhou Rd, Nanjing 210029, Nanjing, China; Cell phone: +13951807457; E-mail address: [zhulingjun@njmu.edu.cn](mailto:zhulingjun@njmu.edu.cn);

Yibing Hua: Department of General Surgery, The First Affiliated Hospital of Nanjing Medical University, 300, GuangZhou Rd, Nanjing 210029, Nanjing, China, Cell phone:+13003423308; E-mail address: 125235225@qq.com.

**Supplementary Table S1.**Pooled ORs and 95% CIs of stratified meta-analysis for *MspI* polymorphism

|  | C/T | | | CC+CT/TT | | | CC/CT+TT | | | CT/TT | | | CC/TT | | |
| --- | --- | --- | --- | --- | --- | --- | --- | --- | --- | --- | --- | --- | --- | --- | --- |
|  | OR | 95% CI | *P* | OR | 95% CI | *P* | OR | 95% CI | *P* | OR | 95% CI | *P* | OR | 95% CI | *P* |
| **Cancer type** |  |  |  |  |  |  |  |  |  |  |  |  |  |  |  |
| EC | 0.94 | 0.71-1.24 | 0.660 | 0.91 | 0.61-1.37 | 0.659 | 0.97 | 0.57-1.65 | 0.905 | 0.91 | 0.61-1.38 | 0.665 | 0.96 | 0.55-1.70 | 0.894 |
| GC | 1.10 | 0.78-1.55 | 0.579 | 1.03 | 0.75-1.41 | 0.860 | 1.33 | 0.59-2.99 | 0.495 | 0.98 | 0.74-1.29 | 0.875 | 1.35 | 0.61-3.01 | 0.463 |
| CC | 1.82 | 1.16-2.86 | **0.010** | 1.67 | 1.06-2.64 | **0.027** | 2.61 | 0.59-11.58 | 0.208 | 1.49 | 0.90-2.49 | 0.122 | 2.47 | 0.61-9.94 | 0.205 |
| **Ethnicity** |  |  |  |  |  |  |  |  |  |  |  |  |  |  |  |
| Asian | 1.06 | 0.83-1.36 | 0.063 | 1.07 | 0.79-1.45 | 0.650 | 1.07 | 0.71-1.63 | 0.737 | 1.05 | 0.78-1.40 | 0.747 | 1.11 | 0.72-1.71 | 0.652 |
| Caucasian | 1.83 | 0.88-3.79 | 0.105 | 1.33 | 1.03-1.73 | **0.031** | 1.86 | 0.24-14.35 | 0.551 | 1.39 | 1.06-1.82 | **0.018** | 1.89 | 0.26-13.93 | 0.532 |
| Mixed | 1.93 | 0.96-3.87 | 0.064 | 1.47 | 0.48-4.45 | 0.496 | 7.05 | 1.75-28.39 | **0.006** | 1.00 | 0.22-4.61 | 0.995 | 5.70 | 1.37-23.60 | **0.016** |
| **Source of control** |  |  |  |  |  |  |  |  |  |  |  |  |  |  |  |
| PB | 1.12 | 0.94-1.33 | 0.211 | 1.10 | 0.92-1.32 | 0.292 | 1.32 | 0.80-2.17 | 0.275 | 1.05 | 0.83-1.32 | 0.706 | 1.28 | 0.81-2.04 | 0.289 |
| HB | 1.69 | 0.31-9.36 | 0.546 | 1.69 | 0.32-9.00 | 0.538 | 1.00 | 0.05-19.42 | 0.998 | 1.64 | 0.38-7.00 | 0.507 | 1.08 | 0.04-27.73 | 0.963 |
| NR | 2.17 | 1.07-4.39 | **0.032** | 2.08 | 1.07-4.01 | **0.030** | 7.90 | 0.42-148.53 | 0.167 | 1.84 | 0.94-3.85 | 0.075 | 8.71 | 0.46-164.16 | 0.148 |
| **Sample size** |  |  |  |  |  |  |  |  |  |  |  |  |  |  |  |
| ≥300 | 0.96 | 0.78-1.18 | 0.699 | 0.98 | 0.74-1.31 | 0.901 | 0.72 | 0.43-1.19 | 0.193 | 1.02 | 0.76-1.39 | 0.883 | 0.69 | 0.40-1.19 | 0.187 |
| <300 | 1.64 | 1.16-2.32 | **0.005** | 1.50 | 1.04-2.16 | **0.032** | 2.39 | 1.20-4.78 | **0.013** | 1.27 | 0.85-1.89 | 0.235 | 2.06 | 1.19-3.57 | **0.010** |
| **Method** |  |  |  |  |  |  |  |  |  |  |  |  |  |  |  |
| PCR-RFLP | 1.64 | 1.16-2.32 | **0.005** | 1.50 | 1.04-2.16 | **0.032** | 2.39 | 1.20-4.78 | **0.013** | 1.27 | 0.85-1.89 | 0.235 | 2.06 | 1.19-3.57 | **0.010** |
| PCR | 0.91 | 0.64-1.29 | 0.600 | 0.93 | 0.64-1.37 | 0.727 | 0.66 | 0.20-2.21 | 0.499 | 0.98 | 0.70-1.35 | 0.88 | 0.64 | 0.16-2.53 | 0.523 |
| Others | 1.24 | 0.99-1.54 | 0.937 | 1.00 | 0.59-1.70 | 0.992 | 0.62 | 0.31-1.23 | 0.17 | 1.04 | 0.56-1.90 | 0.911 | 0.59 | 0.30-1.18 | 0.138 |

EC: esophageal cancer; GC: gastric cancer; CC: colorectal cancer.

**Supplementary Table S2.**Pooled ORs and 95% CIs of stratified meta-analysis for *Ile/Val* polymorphism

|  | G/A | | | GA+GG/AA | | | GG/AA+GA | | | GA/AA | | | GG/AA | | |
| --- | --- | --- | --- | --- | --- | --- | --- | --- | --- | --- | --- | --- | --- | --- | --- |
|  | OR | 95% CI | ***P*** | OR | 95% CI | *P* | OR | 95% CI | *P* | OR | 95% CI | *P* | OR | 95% CI | *P* |
| **Cancer type** |  |  |  |  |  |  |  |  |  |  |  |  |  |  |  |
| EC | 1.36 | 1.19-1.56 | **0.000** | 1.45 | 1.25-1.67 | **0.000** | 1.51 | 1.08-2.10 | **0.015** | 1.42 | 1.22-1.65 | **0.000** | 1.73 | 1.19-2.51 | **0.004** |
| GC | 1.01 | 0.80-1.27 | 0.950 | 0.91 | 0.73-1.12 | 0.363 | 1.59 | 0.75-3.39 | 0.229 | 0.84 | 0.67-1.05 | 0.132 | 1.49 | 0.70-3.15 | 0.299 |
| CC | 1.27 | 1.01-1.61 | **0.043** | 1.34 | 0.97-1.85 | 0.078 | 1.46 | 1.15-1.85 | **0.002** | 1.27 | 0.91-1.79 | 0.164 | 1.45 | 1.02-2.07 | **0.037** |
| **Ethnicity** |  |  |  |  |  |  |  |  |  |  |  |  |  |  |  |
| Asian | 1.21 | 1.07-1.37 | 0.002 | 1.18 | 1.02-1.36 | **0.025** | 1.55 | 1.25-1.92 | **0.000** | 1.10 | 0.95-1.26 | 0.214 | 1.62 | 1.26-2.09 | **0.000** |
| Caucasian | 0.99 | 0.82-1.21 | 0.945 | 1.01 | 0.81-1.24 | 0.953 | 1.05 | 0.46-2.36 | 0.912 | 1.06 | 0.80-1.40 | 0.701 | 1.06 | 0.50-2.24 | 0.874 |
| Mixed | 1.73 | 0.81-3.69 | 0.157 | 2.20 | 0.69-7.00 | 0.181 | 1.95 | 0.71-5.35 | 0.196 | 2.06 | 0.64-6.61 | 0.227 | 4.38 | 0.76-25.18 | 0.098 |
| **Source of control** |  |  |  |  |  |  |  |  |  |  |  |  |  |  |  |
| PB | 1.25 | 0.98-1.58 | 0.068 | 1.37 | 0.99-1.90 | 0.059 | 1.16 | 0.87-1.54 | 0.316 | 1.41 | 1.00-2.00 | 0.052 | 1.31 | 0.89-1.94 | 0.165 |
| HB | 1.23 | 1.05-1.44 | 0.011 | 1.12 | 0.95-1.33 | 0.172 | 1.82 | 1.46-2.26 | **0.000** | 0.99 | 0.86-1.14 | 0.917 | 1.87 | 1.39-2.51 | **0.000** |
| NR | 1.24 | 0.90-1.70 | 0.183 | 1.36 | 0.97-1.90 | 0.071 | 0.96 | 0.34-2.76 | 0.947 | 1.42 | 1.01-2.00 | **0.043** | 1.09 | 0.34-3.47 | 0.886 |
| **Sample size** |  |  |  |  |  |  |  |  |  |  |  |  |  |  |  |
| ≥300 | 1.09 | 1.00-1.20 | 0.063 | 1.08 | 0.96-1.21 | 0.220 | 1.36 | 1.10-1.67 | **0.004** | 1.04 | 0.92-1.18 | 0.516 | 1.36 | 1.09-1.70 | **0.006** |
| <300 | 1.56 | 1.17-2.09 | 0.003 | 1.73 | 1.08-2.79 | **0.023** | 1.92 | 1.26-2.91 | **0.002** | 1.65 | 0.97-2.80 | 0.063 | 2.32 | 1.39-3.86 | **0.001** |
| **Method** |  |  |  |  |  |  |  |  |  |  |  |  |  |  |  |
| PCR-RFLP | 1.32 | 1.03-1.69 | 0.026 | 1.49 | 1.04-2.14 | **0.029** | 1.31 | 0.98-1.76 | 0.073 | 1.55 | 1.04-2.30 | **0.032** | 1.39 | 0.98-1.96 | 0.063 |
| PCR | 1.31 | 1.11-1.55 | 0.002 | 1.26 | 1.05-1.51 | **0.012** | 1.82 | 1.37-2.43 | 0.000 | 1.17 | 1.01-1.35 | **0.034** | 2.05 | 1.44-2.92 | **0.000** |
| Others | 1.06 | 0.86-1.32 | 0.562 | 1.02 | 0.82-1.26 | 0.883 | 1.44 | 0.79-2.61 | 0.232 | 0.97 | 0.79-1.18 | 0.752 | 1.41 | 0.75-2.66 | 0.285 |

EC: esophageal cancer; GC: gastric cancer; CC: colorectal cancer.

**Supplementary Table S3.**Heterogeneity test for *MspI* polymorphism

|  | C/T | | CC+CT/TT | | CC/CT+TT | | CT/TT | | CC/TT | |
| --- | --- | --- | --- | --- | --- | --- | --- | --- | --- | --- |
|  | *I^2^(%)* | *Ph* | *I^2^(%)* | *Ph* | *I^2^(%)* | *Ph* | *I^2^(%)* | *Ph* | *I^2^(%)* | *Ph* |
| **Cancer type** |  |  |  |  |  |  |  |  |  |  |
| EC | 29.90% | 0.232 | 43.60% | 0.183 | 0.00% | 0.714 | 39.50% | 0.199 | 0.00% | 0.496 |
| GC | 59.10% | **0.044** | 21.10% | 0.280 | 58.90% | **0.045** | 0.00% | 0.663 | 53.60% | 0.071 |
| CC | 57.90% | **0.036** | 48.10% | 0.086 | 65.40% | **0.021** | 52.30% | 0.062 | 59.90% | **0.041** |
| **Ethnicity** |  |  |  |  |  |  |  |  |  |  |
| Asian | 58.50% | **0.025** | 53.70% | **0.044** | 22.70% | 0.256 | 46.20% | 0.084 | 21.10% | 0.268 |
| Caucasian | 55.30% | 0.081 | 0.00% | 0.447 | 70.00% | **0.035** | 0.00% | 0.962 | 68.70% | **0.041** |
| Mixed | 48.80% | 0.162 | 72.40% | 0.057 | 0.00% | 0.931 | 82.80% | **0.016** | 0.00% | 0.743 |
| **Source of control** |  |  |  |  |  |  |  |  |  |  |
| PB | 30.00% | 0.178 | 8.00% | 0.369 | 49.10% | **0.047** | 26.40% | 0.21 | 36.40% | 0.127 |
| HB | 91.60% | **0.001** | 89.90% | **0.002** | 69.20% | 0.071 | 86.30% | **0.007** | 74.00% | 0.050 |
| NR | 18.00% | 0.269 | 0.00% | 0.378 | - | - | 0.00% | 0.538 | - | - |
| **Sample size** |  |  |  |  |  |  |  |  |  |  |
| ≥300 | 41.40% | 0.163 | 52.00% | 0.100 | 15.70% | 0.313 | 54.80% | 0.084 | 20.80% | 0.285 |
| <300 | 55.80% | **0.020** | 40.80% | 0.096 | 42.00% | 0.099 | 41.30% | 0.092 | 14.60% | 0.316 |
| **Method** |  |  |  |  |  |  |  |  |  |  |
| PCR-RFLP | 55.80% | **0.020** | 40.80% | 0.096 | 42.00% | 0.099 | 41.30% | 0.092 | 14.60% | 0.316 |
| PCR | 50.50% | 0.155 | 29.60% | 0.233 | 57.70% | 0.124 | 0.00% | 0.363 | 64.90% | 0.092 |
| Others | 63.40% | 0.098 | 76.50% | **0.039** | 0.00% | 0.388 | 80.50% | **0.023** | 0.00% | 0.559 |

*Ph*: *P*-value of Q-test for heterogeneity identification; *I²* index: a quantitative measurement which indicates the proportion of total variation in study estimates that is due to between-study heterogeneity.

**Supplementary Table S4.**Heterogeneity test for *Ile/Val* polymorphism

|  | G/A | | GA+GG/AA | | GG/AA+GA | | GA/AA | | GG/AA | |
| --- | --- | --- | --- | --- | --- | --- | --- | --- | --- | --- |
|  | *I^2^(%)* | *Ph* | *I^2^(%)* | *Ph* | *I^2^(%)* | *Ph* | *I^2^(%)* | *Ph* | *I^2^(%)* | *Ph* |
| **cancer type** |  |  |  |  |  |  |  |  |  |  |
| EC | 22.60% | 0.229 | 0.00% | 0.739 | 32.60% | 0.138 | 0.00% | 0.905 | 37.50% | 0.100 |
| GC | 37.40% | 0.172 | 0.00% | 0.689 | 51.60% | 0.082 | 0.00% | 0.868 | 49.30% | 0.096 |
| CC | 82.30% | **0.000** | 87.60% | **0.000** | 0.00% | 0.472 | 87.80% | **0.000** | 29.10% | 0.186 |
| **ethnicity** |  |  |  |  |  |  |  |  |  |  |
| Asian | 56.80% | **0.002** | 50.20% | **0.010** | 23.50% | 0.182 | 43.60% | **0.028** | 34.60% | 0.080 |
| Caucasian | 0.00% | 0.861 | 0.00% | 0.843 | 28.30% | 0.242 | 17.30% | 0.304 | 19.70% | 0.291 |
| mixed | 91.80% | **0.000** | 94.90% | **0.000** | 13.60% | 0.324 | 94.90% | **0.000** | 56.10% | 0.077 |
| **Source of control** |  |  |  |  |  |  |  |  |  |  |
| PB | 79.80% | **0.000** | 85.50% | **0.000** | 5.20% | 0.394 | 85.60% | **0.000** | 32.90% | 0.136 |
| HB | 59.70% | **0.006** | 43.80% | 0.058 | 2.90% | 0.415 | 17.80% | 0.274 | 28.60% | 0.173 |
| NR | 14.40% | 0.311 | 0.00% | 0.472 | 17.80% | 0.296 | 0.00% | 0.654 | 28.10% | 0.249 |
| **sample size** |  |  |  |  |  |  |  |  |  |  |
| ≥300 | 35.70% | 0.083 | 40.70% | 0.051 | 6.60% | 0.380 | 42.40% | **0.042** | 11.30% | 0.329 |
| <300 | 71.90% | **0.000** | 81.40% | **0.000** | 28.20% | 0.176 | 82.30% | **0.000** | 40.50% | 0.079 |
| **method** |  |  |  |  |  |  |  |  |  |  |
| PCR-RFLP | 80.60% | **0.000** | 86.50% | **0.000** | 22.70% | 0.228 | 87.30% | **0.000** | 36.00% | 0.111 |
| PCR | 44.00% | 0.085 | 28.10% | 0.204 | 0.00% | 0.435 | 2.00% | 0.414 | 14.90% | 0.316 |
| others | 51.10% | 0.056 | 38.90% | 0.132 | 37.20% | 0.145 | 23.70% | 0.248 | 42.10% | 0.110 |

*Ph*: *P-*value of Q-test for heterogeneity identification; *I²* index: a quantitative measurement which indicates the proportion of total variation in study estimates that is due to between-study heterogeneity

**Supplementary Table S5.**Subgroup analyses for adjusted status (Yes or no) and adjusted status especially for smoking history (Yes or no) for Ile/Val polymorphism (GG/AA model).

|  | OR | 95%CI | P |
| --- | --- | --- | --- |
| Adjusted | 1.48 | 1.12-1.96 | **0.005** |
| Non Adjusted | 1.41 | 1.06-1.87 | **0.020** |
| Smoking Adjusted | 1.59 | 1.16-2.18 | **0.004** |
| Non Smoking Adjusted | 1.30 | 1.05-1.62 | **0.019** |

**Supplementary Figure S1:** Forest plot of digestive cancer risk associated with *MspI* polymorphism with adjusted OR and 95%CI (the codominant model CC vs. TT).

**Supplementary Figure S2:** Forest plot of digestive cancer risk associated with *Ile/Val* polymorphism with adjusted OR and 95%CI (the codominant model GG vs. AA).

**Supplementary Figure S3:** Forest plot of digestive cancer risk associated with *MspI* polymorphism after droping the data from Saeed *et al.* 2013[24] (the dominant model CC + CT vs. TT).

**Supplementary Figure S4:** Forest plot of digestive cancer risk associated with *Ile/Val* polymorphism after droping the data from Serafim *et al.* 2008 [47] (the dominant model GA+GG vs. AA).
